# SLC7A7 Downregulation in Monocytes Drives Immunosuppression and Osteosarcoma Progression

**DOI:** 10.1155/ijog/8314828

**Published:** 2026-05-06

**Authors:** Bingjie Jiang, Haoran Zhu, Zhenxing Zhang

**Affiliations:** ^1^ Department of Orthopedics, Taizhou Central Hospital (Taizhou University Hospital), Taizhou, Zhejiang Province, China, tzc.edu.cn; ^2^ Xi′an Jiaotong University Health Science Center, Xi′an, Shaanxi Province, China; ^3^ Department of Maxillofacial Surgery, Taizhou Central Hospital (Taizhou University Hospital), Taizhou, Zhejiang Province, China, tzc.edu.cn

**Keywords:** chemotherapy, metabolism, monocytes, osteosarcoma, SLC7A7

## Abstract

**Background:**

Metabolic reprogramming and the formation of an immunosuppressive tumor microenvironment(TME) are hallmarks of osteosarcoma (OS). However, the metabolic characteristics of OS and its associated immune microenvironment remain largely unknown.

**Methods:**

The single‐cell data were processed for dimensionality reduction and cell‐type annotation by using the Seurat package. Pseudotime analysis and metabolic difference prediction were performed using the SCPA algorithm to predict the metabolic profiles of immune cells. Through integrative analyses using BeyondCell and scMetabolism, three distinct cancer cell subpopulations were identified. Metabolic flux potential and intercellular metabolic communication within each subpopulation were subsequently quantified using METAFlux and Mebocost. Spatial colocalization analysis and intercellular communication prediction were conducted using SpaCET and CellChat. Furthermore, qRT‐PCR and survival analyses were performed on our cohort of OS patients.

**Results:**

Monocytes emerged as the predominant immune cell population within OS tissues, displaying pronounced metabolic reprogramming marked by significant upregulation of glycolysis and tryptophan metabolism. Additionally, three cancer cell subpopulations with distinct chemosensitivity profiles were identified; Subpopulation 2, characterized by high expression of CCNA2, UBE2C, and CENPF, demonstrated significantly reduced sensitivity to methotrexate, doxorubicin, cisplatin, ifosfamide, and etoposide. Moreover, both cancer cells and monocytes function as key metabolic regulators, with glutamine serving as a critical metabolic mediator. Monocytes were predominantly localized in proximity to tumor cells and exhibited activation of signaling pathways such as SPP1 and ICAM. SLC7A7 expression was significantly downregulated in OS tissues, and its expression level was correlated with patient prognosis. Furthermore, monocytes exhibiting SLC7A7 downregulation may display aberrant recruitment patterns and functional deficits, potentially playing a pivotal role in supplying glutamine to OS cells and fostering an immunosuppressive TME.

**Conclusions:**

This study provides a preliminary characterization of the metabolic landscape of OS and its associated immune microenvironment. Targeting SLC7A7‐deficient monocytes may represent promising strategies for enhancing the efficacy of immunotherapy in OS.

## 1. Introduction

Osteosarcoma (OS) is a highly malignant mesenchymal tumor and the most common primary bone malignancy. It is characterized by the direct production of osteoid tissue. OS primarily affects adolescents aged 10–20 years, accounting for approximately 60% of cases, and is a leading cause of cancer‐related death in this age group [1–3]. Long bones, especially the fast‐growing areas such as the distal femur, proximal tibia, and proximal humerus, are the most common sites for OS. Common clinical symptoms include persistent, severe localized pain, progressively enlarged local masses, and restricted movement [1, 4]. Surgical treatment is an important way to treat OS. With the development of medicine, limb‐saving surgery has gradually replaced traditional amputation. However, a significant challenge persists: The majority of OS patients present with micrometastases, predominantly in the lungs, at the time of diagnosis [3, 5]. This contributes to a high recurrence rate and diminished long‐term survival.

Chemotherapy is another cornerstone in the treatment of OS. It plays a crucial role in delaying metastatic progression and improving patient outcomes [6–8]. However, the response rate to single chemotherapy drug is generally low, often below 30%. Therefore, the combination of several chemotherapy drugs can synergistically improve the efficacy. For example, the MAP regimen of methotrexate, doxorubicin, and cisplatin, and the IE regimen of ifosfamide and etoposide have shown good efficacy in the treatment of some OS patients [9–11]. However, there are significant differences in the response of patients to chemotherapy drugs. Further development of methods to predict chemotherapy response, establishment of personalized chemotherapy regimens, and development of new OS treatments (such as immunotherapy and metabolic therapy) are crucial to improving the long‐term survival rate of OS.

In recent years, targeting abnormal tumor metabolism and important regulatory proteins related to metabolic processes has shown promising therapeutic potential by enhancing immune cell activity and facilitating the destruction of tumor cells [12, 13]. The initiation and progression of tumors depend on the adaptive metabolic reprogramming of tumor cells [14]. Dysregulation of glucose, amino acid, and lipid metabolism has been observed in various tumors, including OS, significantly influencing gene expression, cell differentiation, and the development of an immunosuppressive microenvironment [15, 16]. Tumor cells commonly exhibit a preference for glycolysis, known as the Warburg effect, and have a heightened demand for reduced nitrogen [17]. Consequently, tumor cells exhibit altered carbon and nitrogen metabolism, utilize a range of intermediates from the tricarboxylic acid cycle (TCA cycle), and significantly increase nicotinamide adenine dinucleotide phosphate (NADPH) production, thereby sustaining a high proliferative state [17, 18]. In addition, metabolic reprogramming in tumor cells profoundly influences the behavior of immune cells, stromal cells, and other components of the tumor microenvironment (TME). These alterations promote tumor growth and metastasis, suppress antitumor immune responses, and reduce sensitivity to radiotherapy and chemotherapy [19–21]. The efficacy of immunotherapy often depends on the baseline abundance and functional activity of immune cells [22]. Therefore, a comprehensive understanding of the cellular composition and metabolic landscape of OS and its TME is essential for identifying novel targets for metabolic and immunotherapeutic strategies.

The y^+^L amino acid transporter‐1 encoded by solute carrier family 7 member 7 (SLC7A7) serves as the light subunit of a heteromeric amino acid transport protein; it contributes to the formation of cationic amino acid transporters and is primarily responsible for transporting lysine and arginine [23, 24]. Deficiencies in SLC7A7 resulting from gene mutations are closely associated with impaired monocyte function, defective monocyte recruitment, abnormal intracellular arginine accumulation, and lysinuric protein intolerance [25, 26]. Furthermore, downregulating SLC7A7 expression can inhibit both glutamine influx into macrophages and their normal functional activity [27]. Monocytes are a vital component of the immune system and play critical roles in tumor initiation, progression, and therapeutic responses. Aberrant monocytes can differentiate into tumor‐associated macrophages (TAMs), which are closely linked to poor prognosis across multiple tumor types [28]. Tumor cells excessively recruit monocytes by secreting chemokines such as chemokine (C–C motif) ligand 2 (CCL2), thereby promoting angiogenesis and establishing a locally immunosuppressive microenvironment [29].

This study provides a preliminary characterization of metabolic reprogramming in OS and immune cells, particularly monocytes. Monocytes are metabolically regulated by glutamine, which is imported via SLC7A7 and supplied by both tumor cells and the monocytes themselves. Monocytes also play a central role in secreted phosphoprotein 1 (SPP1)‐ and intercellular adhesion molecule 1 (ICAM)‐mediated signaling pathways. In a cohort of OS patients, SLC7A7 expression was significantly downregulated and was positively correlated with patient prognosis. Our findings lay an important foundation for elucidating the metabolic features of OS, enhancing chemotherapy efficacy, and identifying novel immunotherapy therapeutic targets.

## 2. Material and Methods

### 2.1. Dataset Acquisition

OS single‐cell sequencing (scRNA‐seq) data were from GSE162454 in the GEO database (https://www.ncbi.nlm.nih.gov/geo), and normal femoral head tissue was from GSE169396 [30, 31]. A total of 10 tissue samples were included in the study. The OS spatial transcriptomics dataset was obtained from the GEO database (GSE299025) [32].

### 2.2. Single‐Cell Dataset Preprocessing and Dimensionality Reduction Annotation

The scRNA‐seq data of OS tissues were quality controlled using the standard of nFeature_RNA > 200 & nFeature_RNA < 6000 & percent.mt<15, and the scRNA‐seq data of normal tissues were quality controlled using the standard of nFeature_RNA > 200 & nFeature_RNA < 3000 & percent.mt < 15. The SCTransform function in Seurat was used to preprocess the data, and the parameter vars.to.regress = “percent.mt” was set to eliminate the influence of the content of mitochondrial genes in different cells on dimensionality reduction clustering [33]. After the RunPCA function was used to reduce the dimension of the data, Harmony was used to correct the batch effect between different single‐cell samples based on the low‐dimensional data after PCA dimension reduction [34]. The resulting cell clusters were annotated based on marker genes curated in the CellMarker 2.0 database [35]. Finally, dimensionality reduction of the clustered cell populations was visualized using the RunTSNE function.

### 2.3. Prediction of Differences in Cell Metabolism Between Normal Bone Tissue and OS Tissue

The compare_pathways function in SCPA was used to analyze metabolic differences between stromal cells and immune cell populations in both normal and OS tissues [36]. To identify gene sets associated with metabolic pathway differentiation, we employed the combined_metabolic_pathways.csv file provided by the author of the software. The results were then visualized using the plot_rank function.

### 2.4. Pseudotime Analysis and Prediction of Metabolic Differences Between Cells at Different Differentiation Stages

After selecting cell subpopulations from OS tissue, we used the infer_trajectory function in SCPA to perform pseudotime series analysis on the cell subpopulations [35]. In this function, we set the parameter method = ti_slingshot() and used the plot_dimred function to visualize the trajectory. The group_onto_nearest_milestones function can group cells according to the pseudotime value of each cell. After obtaining the grouping, we used compare_pathways to calculate the metabolic differences between different groups and used the plot_rank function to visualize the results.

### 2.5. Identification of Cancer Cells in OS Tissue

The osteoblast population was extracted from the single‐cell data of OS tissue. Cancer cells were identified using the CopyKAT package, applying the copykat function with the parameters ngene.chr = 5 and KS.cut = 0.1 [37].

### 2.6. Prediction of Drug Sensitivity Characteristics of Different Cancer Cell Subpopulations

The bcScore function in Beyondcell was employed to compute the sensitivity scores of individual cancer cells to various drugs [38]. Following this, the bcClusters function was used to categorize the cancer cells based on their sensitivity scores, grouping them into distinct clusters. The bcRanks function was used to determine the priority of candidate drugs in different cancer cell clusters. The bcHistogram function was used to visualize the differences in drug sensitivity between different cancer cell subgroups.

### 2.7. Prediction of Metabolic Differences Between Different Cancer Cell Subpopulations

The scMetabolism package was employed with the VISION algorithm to evaluate and visualize amino acid metabolic pathway scores among cancer cells grouped by drug sensitivity [39].

### 2.8. Calculation of Metabolic Flux Potential of Metabolites in Cancer Tissues

The calculate_avg_exp function in METAFlux was used to create the average expression profile for each cell population, with the parameter n_bootstrap = 60 [40]. The calculate_reaction_score function was then used to calculate the Metabolic Reaction Activity Score (MRAS). Finally, the compute_sc_flux function was used to calculate the metabolic flux potential.

### 2.9. Metabolite Communication Between Different Cell Populations

In Python (Version 3.13.12), the Mebocost package was used to create a Mebocost object using mebocost. create_obj; then, the mebo_obj.infer_commu function was used to infer intercellular metabolic communication [41]. At the same time, we exported the average gene expression value of each type of cell as the input data of COMPASS and used the results returned by COMPASS as the input file of the mebo_obj._ConstainFlux_ function to constrain the prediction results of Mebocost [42]. Finally, the mebo_obj.eventnum_bar and mebo_obj.count_dot_plot functions were used to visualize the prediction results.

### 2.10. Spatial Transcriptomics Analysis

The R package SpaCET was utilized to analyze the spatial transcriptomics data [43]; the create.SpaCET.object function was used to construct a SpaCET object from the spatial transcriptomics data, followed by the application of the SpaCET.deconvolution.matched.scRNAseq function to perform deconvolution of the spatial transcriptomics data based on annotated scRNA‐seq data. Visualization was subsequently carried out using the SpaCET.visualize.spatialFeature function. The SpaCET.CCI.colocalization function was employed to calculate intercellular colocalization, whereas the SpaCET.CCI.LRNetworkScore function was used to predict intercellular interactions. Intercellular communication was analyzed using CellChat [44]. A CellChat object was created using the createCellChat function; after configuring the human intercellular interaction database, network inference was performed using the identifyOverExpressedGenes and identifyOverExpressedInteractions functions. Subsequently, the computeCommunProb and computeCommunProbPathway functions were used to calculate intercellular interactions at both the gene and pathway levels. Finally, the aggregateNet function was applied to aggregate the prediction results and generate visualizations.

### 2.11. Patient Cohort and Quantitative Real‐Time Polymerase Chain Reaction (qRT‐PCR) Validation

For qRT‐PCR analysis and follow‐up studies, fresh surgical specimens and matched adjacent normal tissues were collected from 11 patients with OS treated at our institution between January 2019 and January 2021. Each specimen was snap‐frozen in liquid nitrogen and stored at −80°C for RNA analysis. All paraffin‐embedded specimens from these patients were histologically confirmed by the Department of Pathology at our institution as either OS or normal control tissue. None of the patients received radiotherapy or chemotherapy before surgery. All patients provided written informed consent and had complete follow‐up records. All procedures involving human participants in this study were conducted in accordance with the 1964 Declaration of Helsinki and its subsequent amendments or comparable ethical standards. This study also received approval from the Institutional Review Board of our hospital (Approval No.: 2019KT‐E‐0311). Tissues were cut into small fragments, and total RNA was extracted using TRIzol reagent (Invitrogen). cDNA templates were synthesized using an All‐in‐One miRNA qPCR Kit (GeneCopoeia). Glyceraldehyde‐3‐phosphate dehydrogenase (GAPDH) was used as the internal reference gene. The relative expression levels of SLC7A7 were normalized against control levels using the 2^−*ΔΔ*Ct^ method. The primers used in this experiment were obtained from GeneCopoeia.

### 2.12. Statistical Analysis

All data analyses were performed using R software (Version 4.2.1). Paired‐sample *t*‐tests were used to analyze differences in SLC7A7 expression levels. Survival analysis was conducted using the log‐rank test. One‐way analysis of variance (ANOVA) was employed to analyze differences among multiple groups. A *p* value <0.05 was considered statistically significant.

## 3. Results

### 3.1. Dimensionality Reduction Annotation of Cell Populations in OS Tissue and Normal Bone Tissue

**Figure 1:**
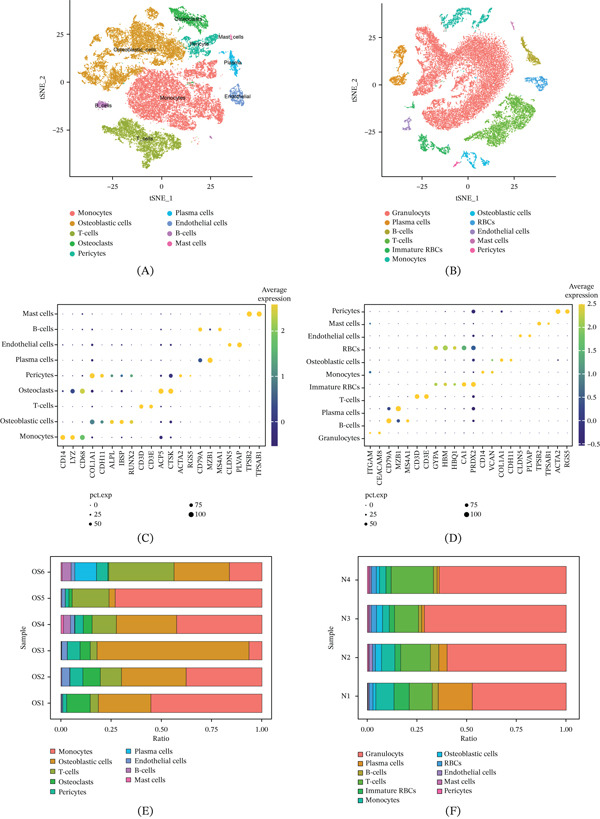
Processing and clustering of single‐cell data from OS and normal femoral head tissues. (A) t‐distributed stochastic neighbor embedding (t‐SNE) dimensionality reduction plot of OS tissue. (B) t‐SNE dimensionality reduction plot of normal femoral head tissue. (C) Key markers of each cell group in OS. (D) Key markers of each cell group in normal femoral head tissue. (E) Proportions of each cell population in OS tissue. (F) Proportions of each cell population in normal femoral head tissue.

After preprocessing and applying dimensionality reduction to the annotations of OS and normal bone tissue single‐cell datasets, we identified key cellular compositions in each tissue type. In the OS TME, nine predominant cell types were observed: monocytes, osteoblastic cells, T‐cells, osteoclasts, pericytes, plasma cells, endothelial cells, B‐cells, and mast cells (Figure 1A). In contrast, normal bone tissue comprised 11 major cell types: granulocytes, plasma cells, B‐cells, T‐cells, immature red blood cells (RBCs), monocytes, osteoblastic cells, mature RBCs, endothelial cells, mast cells, and pericytes (Figure 1B). Figure 1C,D highlights the key markers distinguishing each cell population. For example, CD14 is the main marker of monocytes, whereas marginal zone B1 cell‐specific protein (MZB1) is the main marker of plasma cells. We further quantified the relative proportion of each cell population. The results showed that among the six OS patients, monocytes accounted for the highest proportion in the TME of four OSs. However, in Specimen 3, the osteoblasts of the disease accounted for an overwhelming proportion. The T‐cells in Specimen 6 were the most abundant, followed by tumor cells and monocytes (Figure 1E). Conversely, in the four normal femoral head specimens, granulocytes were the most prevalent cell type. T‐cells still accounted for a relatively abundant proportion among immune cells (Figure 1F).

### 3.2. Metabolic Differences of Immune Cells Between Normal Bone Tissue and OS Tissue

**Figure 2:**
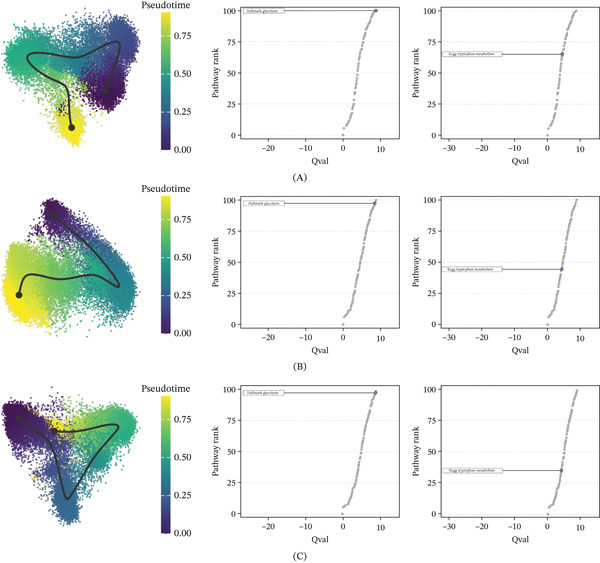
Metabolic differences of immune cells between normal bone tissue and OS tissue. (A) Pseudotime analysis of monocytes. (B) Pseudotime analysis of T‐cells. (C) Pseudotime analysis of B‐cells. The colors from dark to light represent the differentiation direction. The right side shows the rank diagram of pathway differences before and after differentiation.

The metabolic differences of the same types of immune cells in normal tissues and OS tissues were compared. The analysis revealed that glycolysis, reactome phospholipid metabolism, and heme metabolism pathways were significantly enriched in OS‐derived monocytes. Similarly, glycolysis, oxidative phosphorylation, and reactome metabolism of vitamins and cofactors pathways were significantly enriched in OS‐derived T‐cells and B‐cells (Figure S1). In addition, we performed pseudotime analysis on monocytes, T‐cells, and B‐cells, and the results showed that with the differentiation of monocytes, T‐cells, and B‐cells, the two metabolic pathways of glycolysis and tryptophan metabolism were also significantly enhanced (Figure 2A–C).

### 3.3. Metabolic Differences of Stromal Cells Between Normal Bone Tissue and OS Tissue

We further compared the metabolic differences of the same type of stromal cells in normal tissue and OS tissue. The results also showed that there were three metabolic pathways involved, namely glycolysis, reactome collagen biosynthesis and modifying enzymes, and oxidative phosphorylation, which were significantly upregulated in OS‐derived pericytes (Figure S2A). We further extracted OS pericytes and performed pseudotime analysis on this cell population. The results showed that as pericytes differentiated, both glycolysis and tryptophan metabolism pathways were significantly enhanced (Figure S2B). Similarly, we compared the metabolic differences of endothelial cells in normal bone tissue and OS tissue. We found that OS‐derived endothelial cells exhibited significantly higher activities of glycolysis, reactome collagen biosynthesis and modifying enzymes, and oxidative phosphorylation pathways than their normal counterparts (Figure S2C). The pseudotime analysis results also showed that with the differentiation of endothelial cells, the glycolysis and tryptophan metabolism metabolic pathways were significantly enhanced (Figure S2D).

### 3.4. Three Cancer Cell Subpopulations With Different Drug Sensitivity Characteristics Were Identified

**Figure 3:**
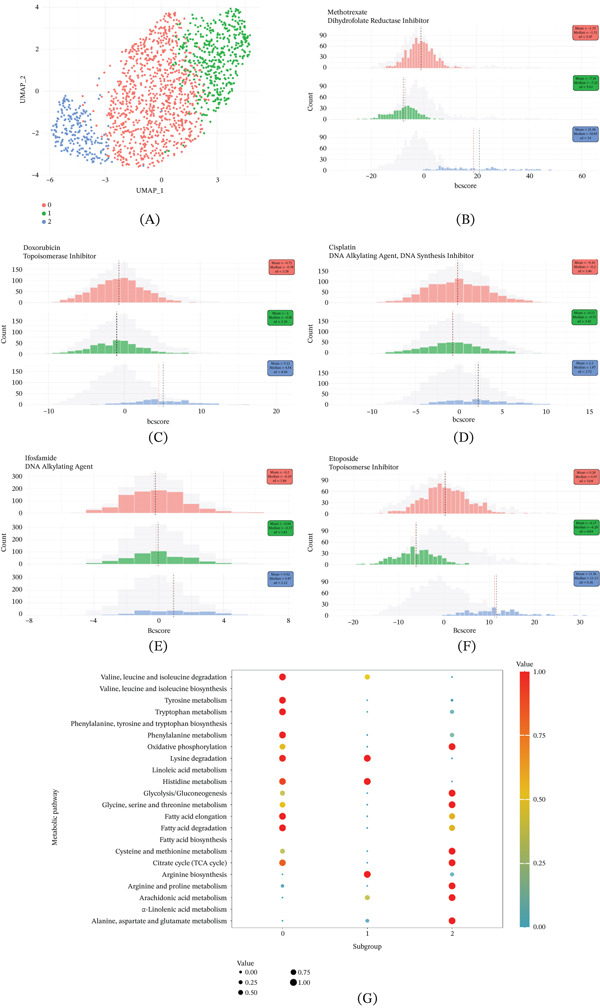
Drug sensitivity characteristics of different OS cancer cell populations. (A) Uniform manifold approximation and projection (UMAP) dimensionality reduction plot of OS cancer cell populations according to drug sensitivity scores. (B–F) Differences in sensitivity of three cancer cell subpopulations to five chemotherapeutic drugs. (G) Metabolic differences of three cancer cell subpopulations.

Considering the pronounced heterogeneity of tumor cells and the importance of chemotherapy as a clinical treatment for OS, we utilized Beyondcell to compute sensitivity scores for each tumor cell against a panel of chemotherapeutic agents. Based on these sensitivity scores, we categorized the cancer cells into subpopulations to better capture the cellular heterogeneity characteristic of OS. Three cancer cell subpopulations with different drug sensitivity characteristics were identified (Figure 3A). Figure 3B–F showed the sensitivity of the three tumor cell subpopulations to five common chemotherapy drugs. The results showed that the cancer cells in Subpopulation 2 were less sensitive to chemotherapy drugs. Additionally, we assessed the metabolic differences across the three subpopulations and observed significant metabolic heterogeneity. For example, cancer cells in Subpopulation 2 exhibited elevated activity in pathways such as oxidative phosphorylation, glycolysis/gluconeogenesis, glycine, serine, and threonine metabolism, the TCA cycle, and arachidonic acid metabolism. However, the metabolic level of lysine degradation in Subpopulation 2 was significantly lower than that in Subpopulations 0 and 1. In contrast, Subpopulation 0 showed significant enrichment in pathways including tryptophan metabolism, tyrosine metabolism, and fatty acid elongation and degradation. Subpopulation 1 was significantly enriched in the arginine biosynthesis pathway (Figure 3G). To further distinguish the three subgroups of cancer cells clinically, we identified the main markers of the three cancer cell subgroups (Table S1). For example, cells in Subgroup 2 highly expressed cyclin A2 (CCNA2), ubiquitin‐conjugating enzyme E2C (UBE2C), and centromere protein F (CENPF).

### 3.5. Differences in Amino Acid Metabolism Among Cell Populations in OS

**Figure 4:**
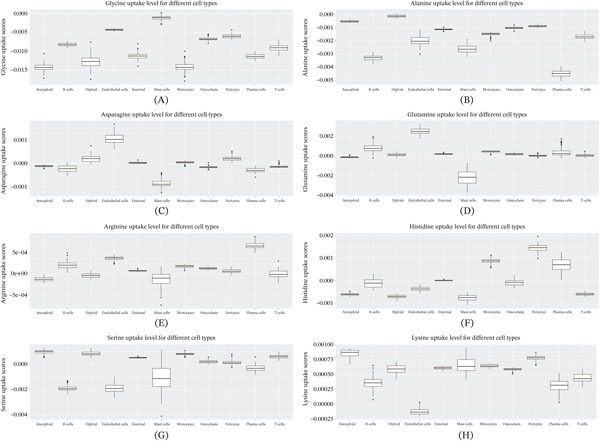
Differences in amino acid metabolism among different cell populations in OS tissue. (A–H) Effects of different OS cell populations on amino acid metabolism. Predicted differences in the metabolism of eight amino acids. Results less than 0 represent absorption and greater than 0 represent release.

We analyzed the metabolic flux potential of 20 amino acids in the human body and identified eight amino acids that exhibited significant changes compared to cancer cells. Tumor cells predicted increased uptake of glycine, alanine, asparagine, glutamine, arginine, and histidine, while simultaneously releasing serine and lysine. Additionally, other cell populations within the TME also exhibited uptake of glycine and alanine, potentially competing with the metabolic demands of cancer cells. With respect to arginine, histidine and glutamine, tumor cells exhibited uptake, whereas monocytes exhibited release. Nearly all cell populations released lysine (Figure 4A–H).

### 3.6. Metabolic Communication Among Cell Populations in OS

**Figure 5:**
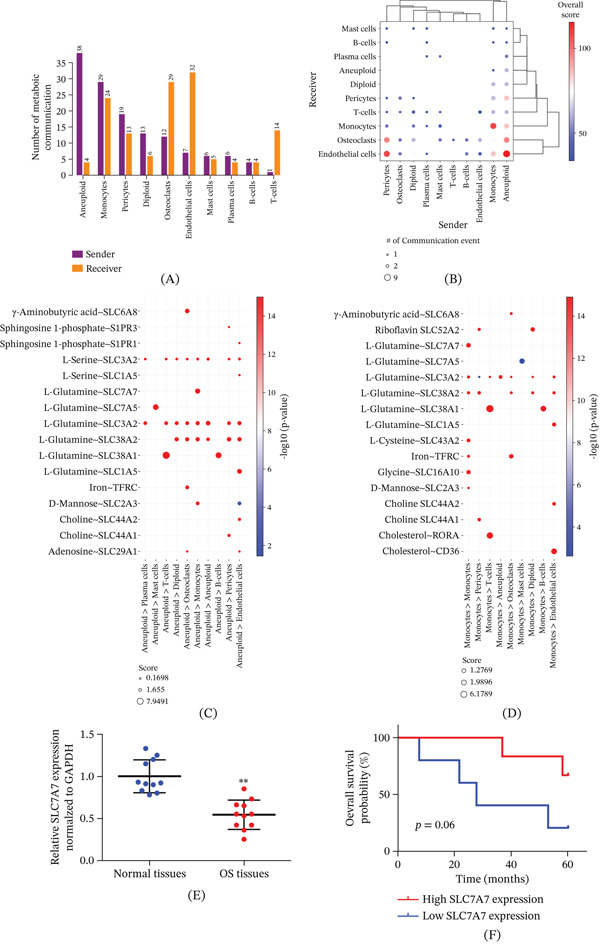
Metabolic communication characteristics between different cell populations in OS tissues. (A) Overview of metabolic communication among various cell populations. (B) Heatmap of metabolic communication among cell populations. (C) Network diagram illustrating tumor cells acting as senders in metabolic communication. (D) Network diagram showing monocytes acting as senders in metabolic communication. (E) The qRT‐PCR results. ∗∗ indicates *p* < 0.01. (F) The survival analysis results.

The Mebocost package was used to further predict the metabolic communication characteristics of each cell population in OS. Figure 5A showed an overview of the predicted intercellular metabolic communication in which each cell population participates as a sender and a receiver. Our analysis revealed that tumor cells rarely act as receivers of metabolic communication; however, they exhibited strong sending activity. Monocytes represented the most abundant cell population involved in metabolic communication. The strength of metabolic communication between each cell population was further quantified. Cancer cells had strong metabolic communication with endothelial cells, osteoclasts, monocytes, and pericytes. In addition, the metabolic communication between monocytes, between endothelial cells and pericytes, and between endothelial cells and monocytes was also strong (Figure 5B). We further defined the metabolic communication in which cancer cells participate as senders. L‐Glutamine was the main metabolic mediator, especially in cancer cells and T, B, and mast cells (Figure 5C). Monocytes receive and transport glutamine via SLC7A7. Considering the high proportion of monocytes and the high intensity of cell communication, we further defined the metabolic communication in which monocytes participate as senders. Again, L‐glutamine stood out as a key mediator. Notably, solute carrier family 3 member 2 (SLC3A2), expressed on cancer cells, may function as a receptor to uptake glutamine secreted by monocytes. Monocytes, meanwhile, receive glutamine and glycine “autocrine” regulatory signals via SLC7A7 and SLC16A10, respectively (Figure 5D). Previous studies show that SLC7A7 is highly expressed in monocytes and is essential for their immune function [24, 25]. Subsequently, we employed qRT‐PCR to examine SLC7A7 expression in OS within our patient cohort. The results revealed that SLC7A7 expression was significantly downregulated in OS tissues compared to adjacent nontumorous tissues (Figure 5E). Furthermore, OS patients with relatively high SLC7A7 expression exhibited a more favorable prognosis (Figure 5F).

### 3.7. Analysis of Cellular Interactions in Spatial Transcriptomics Data

**Figure 6:**
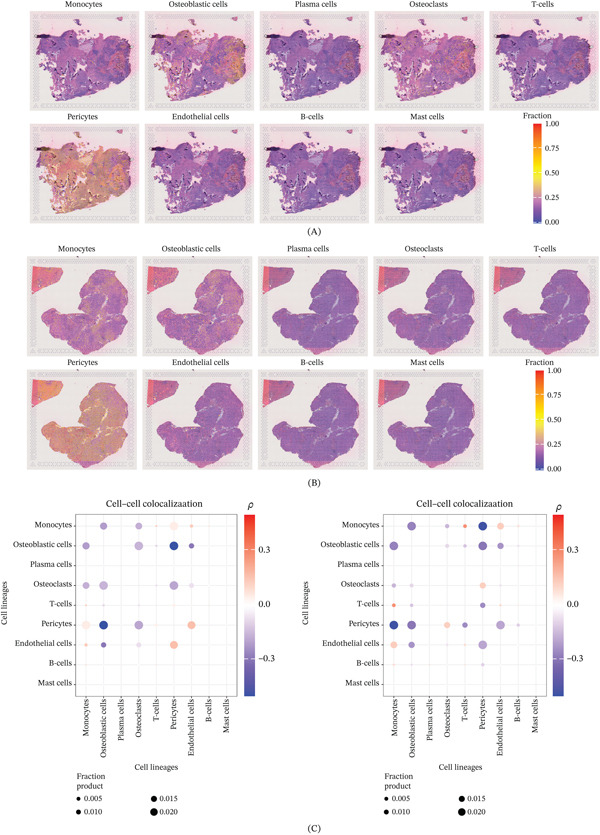
Spatial transcriptomics and cell colocalization analysis. (A–B) Analysis of cell distribution characteristics in two spatial transcriptomics sections. (C) Cell colocalization analysis across the two sections.

**Figure 7:**
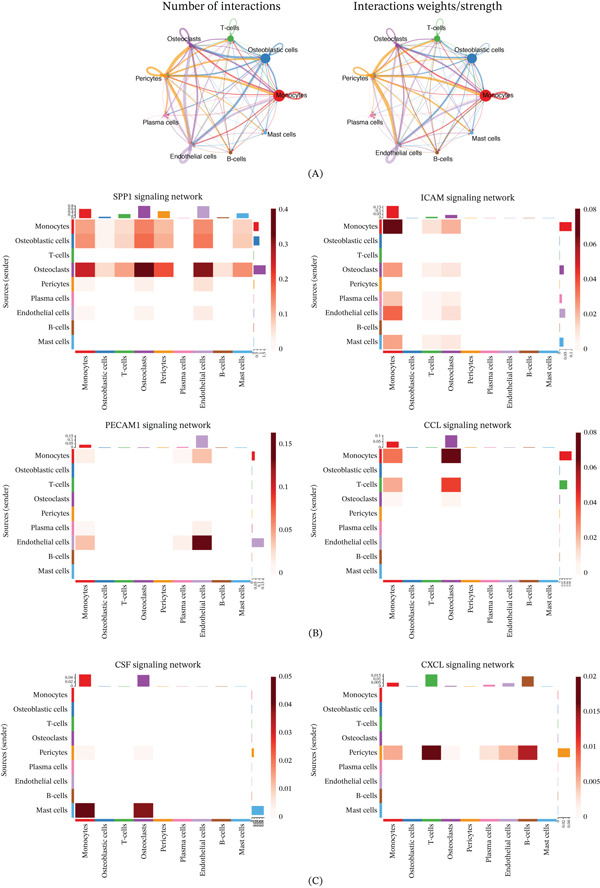
Analysis of chemokine and adhesion factor signaling pathway interactions among TME cells. (A) Overview of intercellular communication. (B, C) The six signaling pathways with the highest interaction intensities. (B) Pathways involving monocytes acting as key senders and (C) pathways involving monocytes acting as key receivers.

We subsequently incorporated spatial transcriptomics data to analyze the distribution and interaction characteristics of cellular populations within the OS TME (Figure 6A,B). The results revealed a mutually exclusive spatial distribution among tumor cells, monocytes, and pericytes (Figure 6C). To elucidate the homing and functional characteristics of various immune cell populations in OS, we further analyzed the impact of chemokines and adhesion molecules on immune cell differentiation and migration (Figure 7A). The six most prominent signaling pathways were identified. Among them, the SPP1 signaling pathway was significantly enriched, with monocytes, tumor cells, and osteoclasts acting as its primary regulators. Monocytes and endothelial cells emerged as the principal regulators of the ICAM and platelet/endothelial cell adhesion molecule 1 (PECAM1) signaling pathways. Furthermore, monocytes and T‐cells were identified as the primary regulators of the CCL signaling pathway. Additionally, mast cells were found to regulate monocyte and osteoclast activity via the colony‐stimulating factor (CSF) signaling pathway, whereas pericytes primarily modulated T‐cells, B‐cells, monocytes, and endothelial cells through chemokine (C–X–C motif) ligand (CXCL) signaling (Figure 7B,C).

## 4. Discussion

OS is a highly malignant primary bone tumor. Due to its invasive, rapid growth and easy hematogenous metastasis, most patients have metastasis at the time of diagnosis. Surgical resection alone is prone to recurrence and metastasis [1, 2, 4]. Chemotherapy is an indispensable part of OS treatment. Traditional adjuvant chemotherapy and preoperative neoadjuvant chemotherapy can cause tumor cell necrosis, control the progression of micrometastases, and facilitate the success of limb‐salvage surgery [9, 45]. However, different patients show obvious heterogeneity in their response to chemotherapy. Predicting chemotherapy responses, developing personalized treatment plans, and elucidating the heterogeneity and biological characteristics of OS are crucial to improving patient outcomes. Adaptive changes in metabolism are a characteristic of malignant tumors. Metabolic intervention has shown encouraging effects in some malignant tumors [46, 47]. However, the metabolic characteristics of OS and the crosstalk within its TME remain poorly understood. This study aims to uncover the metabolic landscape of OS and its TME, thereby identifying potential new protein targets, enhancing immunotherapy strategies, and predicting chemotherapy responses. We included single‐cell data from six OSs and four normal bone tissues, and through dimensionality reduction and annotation, we clarified the cell population composition of OS and normal bone tissue. Monocytes were an abundant cell population in the OS microenvironment. Furthermore, we found that RBC cells in normal tissues are more abundant than in tumor tissues, which is closely related to the active proliferation and hypoxia of OS.

Hypoxia is a common feature of solid tumors [48]. Under hypoxic conditions, cancer cells rely heavily on glycolysis to sustain their growth, characterized by increased glucose uptake and excessive lactate production. In addition, hypoxia can also cause the release of growth factors such as transforming growth factor‐*β* (TGF‐*β*) and epidermal growth factor (EGF), promoting the recruitment and transformation of cancer‐associated fibroblasts and M2 macrophages. These changes collectively promote cancer cell survival and resistance to chemotherapy and immunotherapy [49]. Our results showed that both stromal and immune cells in OS showed significantly enhanced glycolysis. In addition, as stromal cells and immune cells differentiated, the intensity of their glycolytic pathways continued to increase. This suggests that the highly hypoxic environment in tumors may play an important role in the plasticity of the TME and the differentiation of related cell populations.

Tryptophan metabolism, driven primarily by the enzymes indoleamine‐2,3‐dioxygenase 1 (IDO1) and tryptophan 2,3‐dioxygenase (TDO), has garnered significant interest as a potential target for novel immunotherapy strategies [13, 50]. These enzymes catalyze the breakdown of tryptophan into kynurenine, which subsequently activates the aryl hydrocarbon receptor, promoting cancer cell motility and strongly suppressing the antitumor activity of immune cells such as T‐cells, macrophages, and dendritic cells [51]. In addition, studies have found that interleukin 4 induced 1 (IL4I1) consumes tryptophan to produce indole‐3‐pyruvate, which significantly enhances tumor cell motility and inhibits tumor immunity in an aryl hydrocarbon receptor‐dependent pathway [52]. Recent studies have also confirmed that the tryptophan metabolic pathway can mediate the antiferroptosis activity of tumor cells [53]. In this study, we observed that tryptophan metabolism was significantly upregulated during the differentiation of stromal and immune cells, suggesting a key role for tryptophan in reshaping cellular populations within the OS TME and in mediating immune cell dysfunction. Targeting proteins related to tryptophan metabolism may enhance antitumor immune effects.

Considering that chemotherapy is an indispensable treatment for OS, in this study, we intend to develop a potential method for predicting the efficacy of OS chemotherapy. Tumor cells exhibit significant heterogeneity, making it challenging to summarize their characteristics through direct clustering. We first identified three cancer cell subpopulations with different drug sensitivity characteristics based on the drug sensitivity score, among which the cancer cells in Subpopulation 2 were less sensitive to chemotherapy drugs. We further identified the main marker proteins of the three cancer cells to provide a reference for the clinical prediction of chemotherapy effects. In short, through immunohistochemical staining of the lesion tissue after surgical resection, if CCNA2, UBE2C, and CENPF are highly expressed, it may be considered that there is a higher proportion of subpopulation 2 cancer cells, which may associated with chemoresistance and a poorer response to MAP/IE chemotherapy, suggesting that combination with other therapeutic strategies may be needed. On the contrary, if the cancer section highly expresses adenine phosphoribosyltransferase (APRT) or activating transcription factor 3 (ATF3), it is inferred that there is a higher proportion of subpopulation 0 and subpopulation 1 cancer cells, suggesting a better response to MAP/IE chemotherapy. Furthermore, our analysis of metabolic differences among the three subpopulations revealed significant heterogeneity in metabolic pathways, implying that metabolic changes may play a critical role in determining tumor sensitivity to chemotherapy.

In addition to tumor cells, solid tumor tissues also harbor a variety of stromal cells, immune cells, and other components that together create a complex microenvironment characterized by extensive cellular crosstalk. This microenvironment plays a pivotal role in tumor progression, including rapid growth, metastasis, immune evasion, therapy resistance, and tolerance to radiotherapy and chemotherapy [54]. Therefore, we further clarified the metabolic communication characteristics of each cell population in OS. Tumor cells were confirmed to be the core of regulation, mainly as senders to regulate the metabolism of other cell populations. Monocytes constituted an extremely high proportion of OS and were involved in most cell communication. Abnormal proportions and functional defects in monocytes can significantly suppress adaptive immunity, which may be closely related to the progression and metastasis of OS [23, 24]. Furthermore, glutamine, as the most important metabolic regulatory mediator, has been confirmed in previous studies to be an important source of energy and intermediate metabolites for rapidly proliferating cancer cells and immune cells [12, 55, 56]. Most cancer cells are “addicted” to glutamine and usually consume glutamine at a rate higher than glucose [57]. Glutamine metabolism in cancer cells also affects the recruitment and activation of myeloid cells. Studies have shown that inhibiting glutaminase can limit the infiltration of myeloid suppressor cells (MDSCs) in tumors and promote the repolarization of TAMs to the immunostimulatory M1‐like phenotype [58]. SLC7A7 is highly expressed on the surface of normal monocytes. Our findings indicated that monocytes predominantly rely on SLC7A7 to uptake glutamine produced by both tumor cells and autocrine sources. In contrast, tumor cells primarily utilized SLC3A2 to acquire glutamine supplied by monocytes. Spatial transcriptomic analysis indicated that monocytes within OS are closely associated with the SPP1 and ICAM signaling pathways. By interacting with its receptors such as CD44 and integrins, SPP1 activates key signaling cascades, including the phosphatidylinositol 3‐kinase (PI3K)/protein kinase B (PKB) and mitogen‐activated protein kinase (MAPK)/extracellular signal‐regulated kinase (ERK) pathways, and plays a critical role in regulating tumor stem cell properties, promoting M2 polarization of TAMs, and fostering an immunosuppressive TME [59, 60]. ICAM‐1 is a key transmembrane adhesion molecule in the immunoglobulin superfamily, broadly expressed on the surface of diverse cell types, including endothelial and immune cells. The aberrant activation of the ICAM signaling pathway is closely linked to immunosuppression and tumor metastasis [61, 62]. These findings suggested that monocytes in the OS microenvironment are functionally impaired and exhibit immunosuppressive characteristics. Furthermore, validation within our enrolled OS patient cohort confirmed that SLC7A7 expression is significantly downregulated in OS, a phenomenon positively correlated with patient prognosis. Consequently, we believed that monocytes with downregulated SLC7A7 are functionally impaired; not only do they furnish OS cells with abundant nutrients, but they also drive the aberrant recruitment of additional monocytes and facilitate the establishment of an immunosuppressive microenvironment. It remains to be determined whether overexpression of SLC7A7 can enhance the therapeutic efficacy of OS treatment by inhibiting glutamine uptake in tumor cells.

## 5. Conclusion

In summary, this study investigated the metabolic characteristics of OS and monocytes. Through the analysis and comparison of single‐cell datasets, we preliminarily confirmed the hypoxic environment and metabolic alterations in OS. Tryptophan metabolism may play an important role in the progression of OS. Additionally, we identified three distinct cancer cell subpopulations characterized by varying levels of drug sensitivity and unique metabolic profiles. Based on this, we proposed a potential method for predicting chemotherapy response. Ten genes—including CCNA2, UBE2C, and CENPF—emerged as candidate markers for assessing OS′s sensitivity to five commonly used chemotherapeutic agents. Furthermore, we predicted the complex metabolic crosstalk between tumor cells and the cellular populations within their microenvironment, identifying glutamine as a critical metabolic mediator in OS. We proposed that monocytes with low SLC7A7 expression play a dual role: They conserve glutathione to support cancer cell growth while simultaneously recruiting a population of functionally impaired monocytes, thereby promoting an immunosuppressive TME through signaling pathways such as SPP1 and ICAM. This study may facilitate the clinical development of novel therapeutic targets, enhance the efficacy of chemotherapy, immunotherapy, and metabolic therapy, and ultimately improve the prognosis for patients with OS. However, this study has some limitations. First, it relies primarily on single‐cell data and bioinformatics analyses, without validation through laboratory experiments, making it a preliminary exploration. Second, the scarcity of available single‐cell datasets and limited clinical samples for OS may affect the robustness of our conclusions. Further validation using larger sample sizes and experimental studies is essential to strengthen these findings. Third, it is essential to further isolate and culture OS‐derived monocytes to validate their transcriptional and metabolic reprogramming.

**Nomenclature**
TME: tumor microenvironment
OS: osteosarcoma
TCA cycle: tricarboxylic acid cycle
NADPH: nicotinamide adenine dinucleotide phosphate
SLC7A7: solute carrier family 7 member 7
TAMs: tumor‐associated macrophages
CCL2: chemokine (C–C motif) ligand 2
SPP1: secreted phosphoprotein 1
ICAM: intercellular adhesion molecule 1
scRNA‐seq: single‐cell sequencing
MRAS: metabolic reaction activity score
qRT‐PCR: quantitative real‐time polymerase chain reaction
GAPDH: glyceraldehyde‐3‐phosphate dehydrogenase
ANOVA: analysis of variance
RBCs: red blood cells
MZB1: marginal zone B1 cell‐specific protein
CCNA2: cyclin A2
UBE2C: ubiquitin‐conjugating enzyme E2C
CENPF: centromere protein F
SLC3A2: solute carrier family 3 member 2
PECAM1: platelet/endothelial cell adhesion molecule 1
CSF: colony‐stimulating factor
CXCL: chemokine (C–X–C motif) ligand
TGF‐*β*
: transforming growth factor‐*β*
EGF: epidermal growth factor
IDO1: enzymes indoleamine‐2,3‐dioxygenase 1
TDO: tryptophan 2,3‐dioxygenase
IL4I1: interleukin 4 induced 1
APRT: adenine phosphoribosyltransferase
ATF3: activating transcription factor 3
MDSCs: myeloid suppressor cells
PI3K: phosphatidylinositol 3‐kinase
PKB: protein kinase B
MAPK: mitogen‐activated protein kinase
ERK: extracellular signal‐regulated kinase
t‐SNE: t‐distributed stochastic neighbor embedding
UMAP: uniform manifold approximation and projection

## Author Contributions

Conceptualization, Bingjie Jiang, Haoran Zhu, and Zhenxing Zhang; formal analysis, Bingjie Jiang and Haoran Zhu; funding acquisition, Zhenxing Zhang; project administration, Zhenxing Zhang; supervision, Zhenxing Zhang; writing – original draft, Bingjie Jiang; writing – review and editing, Bingjie Jiang and Zhenxing Zhang. Bingjie Jiang and Haoran Zhu have contributed equally to this work and share first authorship.

## Funding

This study was supported by the Taizhou Social Development Science and Technology Project, 23ywa20.

## Disclosure

After using Grammarly, the authors reviewed and edited the content as needed and take full responsibility for the content of the publication. All authors have approved the manuscript and agree with its submission to the International Journal of Genomics.

## Ethics Statement

All patients provided written informed consent and had complete follow‐up records. All procedures involving human participants in this study were conducted in accordance with the 1964 Declaration of Helsinki and its subsequent amendments or comparable ethical standards. This study also received approval from the Institutional Review Board of our hospital (2019KT‐E‐0311).

## Conflicts of Interest

The authors declare no conflicts of interest.

## Supporting information


**Supporting Information** Additional supporting information can be found online in the Supporting Information section. Table S1: Characteristic gene expressions of three cancer cell subpopulations with different drug sensitivity characteristics. Figure S1: Metabolic differences of immune cells between normal bone tissue and OS tissue. Rank plot of pathway differences of monocytes (A), T‐cells (B), and B‐cells (C) in normal bone tissue and OS tissue. Figure S2: Metabolic differences in stromal cells between normal bone tissue and OS tissue. (A) Rank plot depicting pathway differences in pericytes from normal bone tissue and OS tissue. (B) Pseudotime analysis of pericytes. Colors from dark to light represent the direction of differentiation. The right side shows the rank plot of pathway differences before and after differentiation. (C) Rank plot showing pathway differences in endothelial cells from normal bone tissue and OS tissue. (D) Pseudotime analysis of endothelial cells. The colors from dark to light represent the direction of differentiation. The right side shows the rank plot of the pathway differences before and after differentiation.

## Data Availability

The data that support the findings of this study are available from the corresponding author upon reasonable request.
