# Supplementary material for: SLC7A7 Downregulation in Monocytes Drives Immunosuppression and Osteosarcoma Progression
Source: Int J Genomics. 2026 May 6;2026:8314828. doi: 10.1155/ijog/8314828 (PMC13147929; doi:10.1155/ijog/8314828)
Supplement: Supplementary file 1 — Supporting Information Additional supporting information can be found online in the Supporting Information section. Table S1: Characteristic gene expressions of three cancer cell subpopulations with different drug sensitivity characteristics. Figure S1: Metabolic differences of immune cells between normal bone tissue and OS tissue. Rank plot of pathway differences of monocytes (A), T‐cells (B), and B‐cells (C) in normal bone tissue and OS tissue. Figure S2: Metabolic differences in stromal cells between normal bone tissue and OS tissue. (A) Rank plot depicting pathway differences in pericytes from normal bone tissue and OS tissue. (B) Pseudotime analysis of pericytes. Colors from dark to light represent the direction of differentiation. The right side shows the rank plot of pathway differences before and after differentiation. (C) Rank plot showing pathway differences in endothelial cells from normal bone tissue and OS tissue. (D) Pseudotime analysis of endothelial cells. The colors from dark to light represent the direction of differentiation. The right side shows the rank plot of the pathway differences before and after differentiation. [file IJOG-2026-8314828-s001.docx]

**Table S1. Characteristic gene expressions of three cancer cell subpopulations with different drug sensitivity characteristics.**

| Subgroup | Genes | | | | |
| --- | --- | --- | --- | --- | --- |
| 0 | APRT | IFITM5 | GSN | LY6E | CREB3L1 |
|  | COL11A2 | IBSP | GRP | GZMA | FGFBP2 |
| 1 | ATF3 | FOS | JUNB | CYR61 | MAFF |
|  | DDIT3 | BTG2 | SAT1 | ZFAND2A | MEG3 |
| 2 | CCNA2 | UBE2C | CENPF | H2AFZ | CKS1B |
|  | KPNA2 | TOP2A | CCNB1 | PTTG1 | HIST1H4C |


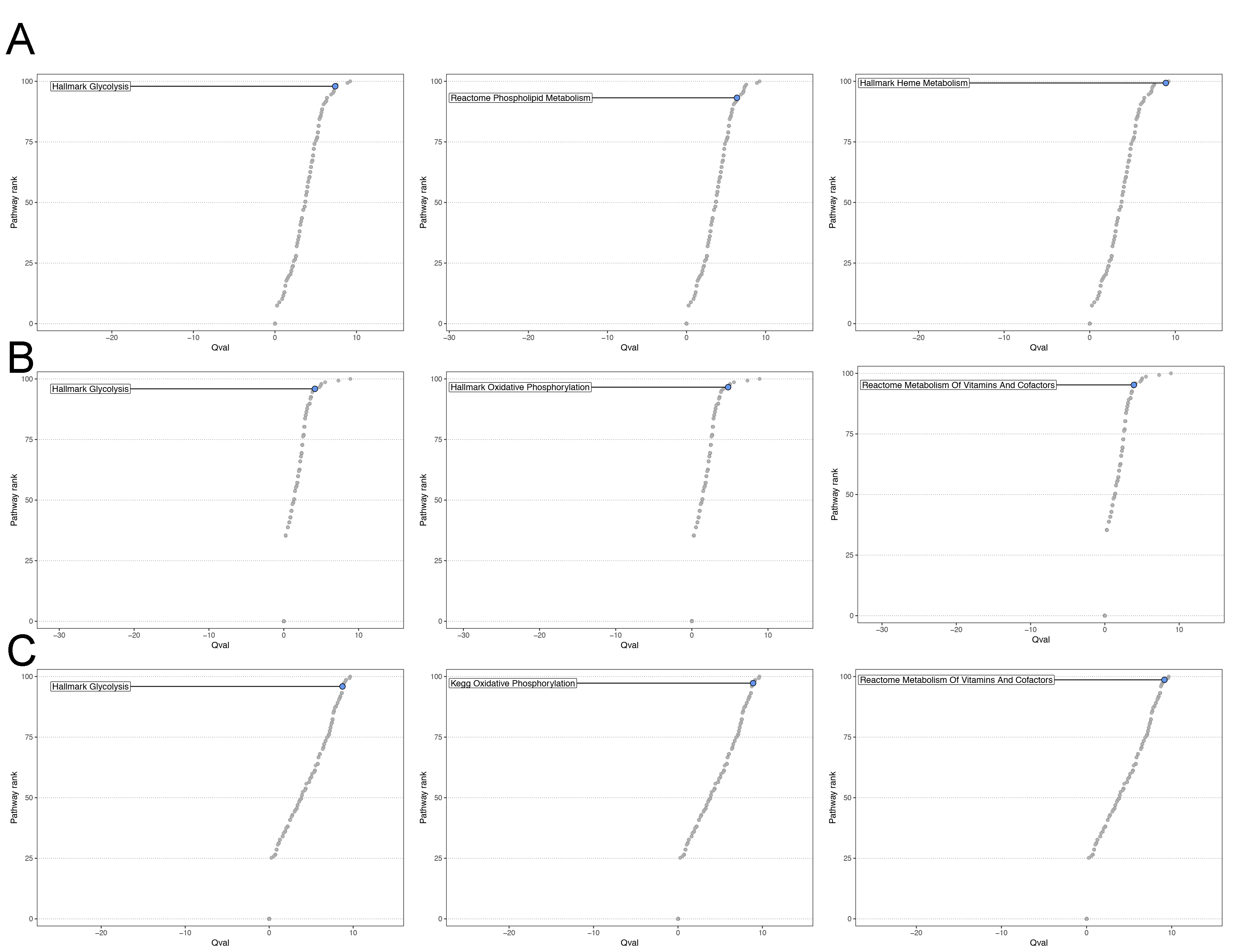


**Figure S1. Metabolic differences of immune cells between normal bone tissue and OS tissue.** Rank plot of pathway differences of monocytes (A), T-cells(B), and B-cells (C) in normal bone tissue and OS tissue.


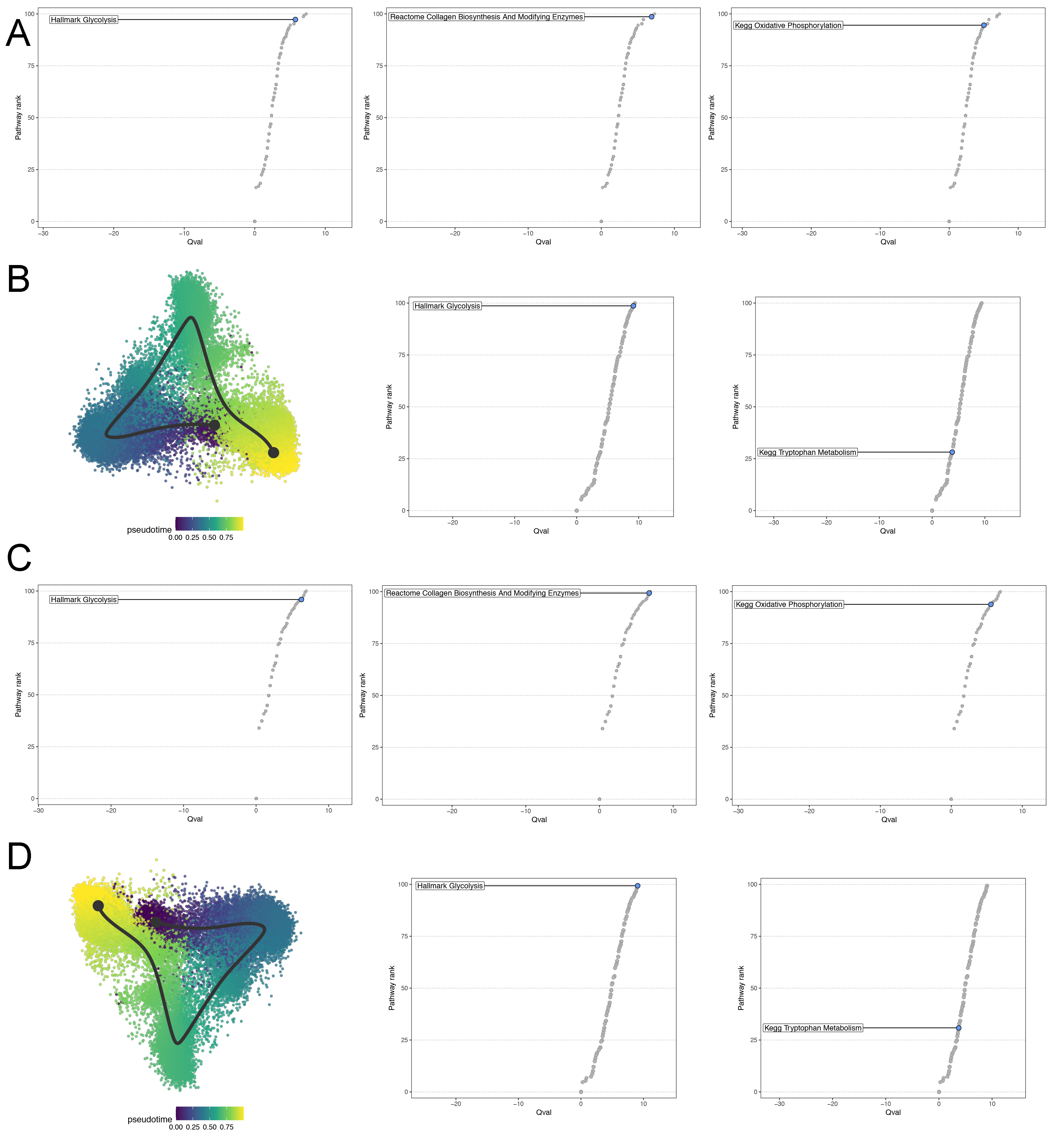


**Figure S2.​ Metabolic differences in stromal cells between normal bone tissue and OS tissue.** (A) Rank plot depicting pathway differences in pericytes from normal bone tissue and OS tissue. (B) Pseudotime analysis of pericytes. Colors from dark to light represent the direction of differentiation. The right side shows the rank plot of pathway differences before and after differentiation. (C) Rank plot showing pathway differences in endothelial cells from normal bone tissue and OS tissue. (D) Pseudotime analysis of endothelial cells. The colors from dark to light represent the direction of differentiation. The right side shows the Rank plot of the pathway differences before and after differentiation.
